# Endocrine meets hepatic: the thyroid–liver axis in health and disease—an overview

**DOI:** 10.1007/s10354-026-01180-5

**Published:** 2026-09-14

**Authors:** Hagar Abdelsamad, Peter Mikosch

**Affiliations:** 1 University Hospital Krems, Department of Internal Medicine 2, Krems, Austria; 2 Department of Internal Medicine 2, General Hospital Mistelbach, Liechtensteinstraße 67, 2130 Mistelbach, Austria; 3 Karl Landsteiner Institut für Inflammation und Stoffwechsel, Mistelbach, Austria; 4 Teaching Unit, Medizinische Universität Wien, Vienna, Austria

**Keywords:** Thyroid–liver axis, Thyroid hormones, Metabolic dysfunction-associated steatotic liver disease, Thyromimetics, Non-thyroidal illness syndrome, Schilddrüsen-Leber-Achse, Schilddrüsenhormone, Mit metabolischer Dysfunktion assoziierte steatotische Lebererkrankung, Thyromimetika, Non-Thyroidal Illness Syndrome

## Abstract

The thyroid and the liver share a complex and bidirectional relationship, where thyroid hormones significantly influence hepatic metabolism and vice versa. Through the hypothalamic–pituitary–thyroid (HPT) axis, thyroid hormones regulate lipid, glucose, and protein metabolism, while the liver plays a pivotal role in thyroid hormone metabolism, transport, and conversion. Disturbances of the thyroid–liver metabolic axis can have significant clinical consequences. Examples include the association between hypothyroidism and metabolic dysfunction-associated steatotic liver disease (MASLD), as well as the development of non-thyroidal illness syndrome (NTIS) in patients with chronic liver disease. In addition, autoimmune thyroid and liver diseases may coexist due to shared immunological mechanisms. Recent advances, including the approval of resmetirom by the U.S. Food and Drug Administration (FDA) in 2024 and its subsequent approval by the European Medicines Agency (EMA) in 2025, highlight the therapeutic potential of thyromimetics for treatment of metabolic-associated steatohepatitis (MASH) and support further exploration of thyroid hormone signaling as a therapeutic target in liver disease. Additionally, emerging research explores the utility of thyroid biomarkers as prognostic tools, offering new prospects to enhance screening and optimize treatment strategies. This review synthesizes insights from over 40 research articles, focusing on the molecular mechanisms underpinning the thyroid–liver axis and evaluating its clinical and therapeutic implications. These findings underscore the vital role of thyroid hormones in liver health and suggest innovative approaches for managing thyroid-related liver disorders.

## Abstract (de)

Die Schilddrüse und die Leber stehen in einer komplexen, bidirektionalen Wechselwirkung, bei der Schilddrüsenhormone den hepatischen Stoffwechsel wesentlich beeinflussen – und umgekehrt. Über die hypothalamisch-hypophysäre Schilddrüsenachse (HPT-Achse) regulieren Schilddrüsenhormone den Lipid‑, Glukose- und Proteinstoffwechsel, während die Leber eine zentrale Rolle in dem Metabolismus, Transport und der Umwandlung dieser Hormone spielt. Störungen dieser Interaktion können klinisch bedeutsame Folgen haben. Beispiele hierfür sind der Zusammenhang zwischen Hypothyreose und der mit metabolischer Dysfunktion assoziierten steatotischen Lebererkrankung (MASLD) sowie die Entwicklung des Non-Thyroidal Illness Syndrome (NTIS) bei Patienten mit chronischen Lebererkrankungen. Darüber hinaus können autoimmune Schilddrüsen- und Lebererkrankungen aufgrund gemeinsamer immunologischer Mechanismen gehäuft gemeinsam auftreten. Jüngste Entwicklungen, darunter die Zulassung durch die U.S. Food and Drug Administration (FDA) von Resmetirom im Jahr 2024 sowie die anschließende Zulassung durch die Europäische Arzneimittel-Agentur (EMA) im Jahr 2025, unterstreichen das therapeutische Potenzial von Thyromimetika zur Behandlung der mit metabolischer Dysfunktion assoziierten Steatohepatitis (MASH) und unterstützen die weitere Erforschung der Schilddrüsenhormon-Signalwege als therapeutisches Ziel bei Lebererkrankungen. Darüber hinaus untersucht die aktuelle Forschung die Bedeutung von Schilddrüsenbiomarkern als prognostische Instrumente, die neue Möglichkeiten für Screening, Risikostratifizierung und Therapieoptimierung eröffnen könnten. Im vorliegenden Übersichtsartikel wurden Erkenntnisse aus mehr als 40 wissenschaftlichen Publikationen zusammengefasst und die molekularen Mechanismen der Schilddrüsen-Leber-Achse sowie deren klinische und therapeutische Bedeutung beleuchtet. Die vorliegenden Daten unterstreichen die zentrale Rolle von Schilddrüsenhormonen für die Lebergesundheit und weisen auf innovative Ansätze zur Behandlung schilddrüsenassoziierter Lebererkrankungen hin.

## Introduction

The thyroid and the liver share a tightly regulated relationship, which is vital for maintaining overall metabolism, normal organ growth, and development. This interaction operates under the control of the hypothalamic–pituitary–thyroid (HPT) axis, where thyroid-stimulating hormone (TSH), secreted by the pituitary gland, drives thyroid hormone production.

The basal metabolic rate of all cells, including hepatocytes, is regulated by thyroxine (T4) and triiodothyronine (T3), which modulates hepatic function. Conversely, the liver plays a key role in modulating thyroid hormone activity through conversion, conjugation, and transport [1, 2].

Any metabolic dysfunction of the thyroid hormone (TH)/thyroid hormone receptor (THR) axis can lead to various systemic disturbances affecting growth, skeletal development, cardiovascular health, cognitive function, gastrointestinal function, obesity, metabolic dysfunction, and even cancer [3].

Given the growing burden of chronic liver disease in Europe, recent initiatives such as the second European Association for the Study of the Liver (EASL)–Lancet Commission have emphasized the importance of prevention, early detection, and sustainable liver health strategies [4].

This narrative review is based on a literature search performed using PubMed, Google Scholar, and relevant scientific journals. The review included publications on the physiology of the thyroid–liver axis, the underlying pathophysiological mechanisms, clinical manifestations, metabolic dysfunction-associated steatotic liver disease (MASLD; formerly known as NAFLD), thyroid hormone signaling, thyromimetic agents, and hepatocellular carcinoma. Recent clinical studies, meta-analyses, randomized controlled trials, and landmark publications relevant to the topic were prioritized in order to provide an overview of current evidence and highlight potential clinical implications as well as emerging therapeutic perspectives.

## Physiology of the thyroid and its effect on hepatic metabolism

**Fig. 1:**
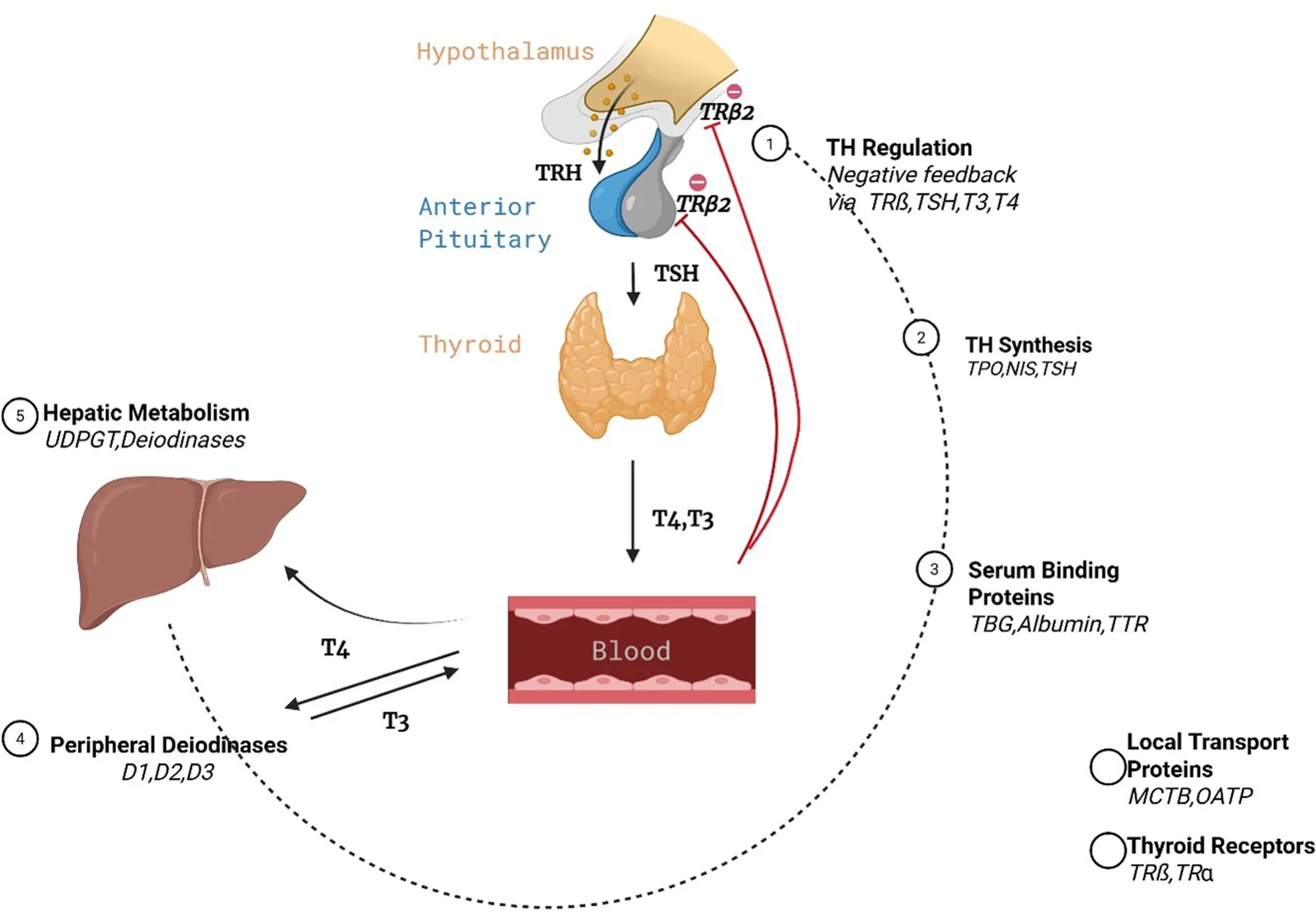
Regulation and metabolism of thyroid hormones. (1) Negative feedback by T3/T4 via THR‑β on the hypothalamus and anterior pituitary; (2) TSH-stimulated synthesis of T4/T3 via TPO and NIS in the thyroid gland; (3) serum transport bound to TBG, albumin, and TTR; (4) peripheral deiodination by D1, D2, and D3; (5) hepatic metabolism via deiodinase and UDP-glucuronosyltransferase (UDPGT). *TH* thyroid hormones, *TRH* thyrotropin-releasing hormone, *TSH* thyroid-stimulating hormone, *TPO* thyroid peroxidase, *NIS* sodium/iodide symporter, *TBG* thyroxine-binding globulin, *TTR* transthyretin, *D1/2/3* = deiodinases; *UDPGT* UDP-glucuronosyltransferase, *OATP* organic anion-transporting polypeptide

Thyrotropin-releasing hormone (TRH) stimulates the anterior pituitary gland to secrete thyroid-stimulating hormone (TSH), which in turn stimulates the thyroid gland to produce thyroid hormones. Thyroid-stimulating hormone secretion is regulated by the circulating levels of free T3 and T4 by negative feedback regulation. While T4 is the predominant hormone secreted by the thyroid, the majority of T3 is derived from conversion of T4 to T3 in peripheral tissues by thyroxine deiodinase ([5]; Fig. 1). T3 plays a critical role in regulating various physiological processes, including cardiovascular function, metabolism, and overall growth and development [6].

Thyroid hormones are essential for various hepatic metabolic activities and biochemical pathways [7]. Regarding lipid metabolism, thyroid hormones promote fatty acid uptake and stimulate de novo lipogenesis through upregulation of enzymes like acetyl-CoA carboxylase and fatty acid synthase [8, 9]. Additionally, they stimulate cholesterol synthesis via the mevalonate pathway, promote LDL uptake, and enhance cholesterol excretion via reverse transport mechanisms [10]. In connection with glucose homeostasis, thyroid hormones facilitate glycogenolysis and gluconeogenesis, particularly during fasting [6].

## Liver physiology and its effect on thyroid metabolism

The liver is the primary site for conversion of T4 to its active form T3. Thyroid hormones T4 and T3 enter hepatic cells via specialized transporters, such as monocarboxylate transporter 8 (MCT8) and organic anion-transporting polypeptide 1B1 (OATP1B1) [6, 7]. Within the liver, T4 is converted into active T3 by the enzymes deiodinase 1 (DIO1) and deiodinase 2 (DIO2), while deiodinase 3 (DIO3) inactivates T4 and T3 by converting them to reverse T3 (rT3) and diiodothyronine. The major enzyme in the liver is DIO1, which accounts for approximately 30%–40% of the extrathyroidal production of T3. T3 exerts its effects by binding to thyroid hormone receptors (THR‑α and THR-β), which regulate gene transcription and thereby influence cellular metabolic activity [3].

## Pathophysiology of the thyroid–liver interaction

Any dysregulation in the thyroid–liver axis can result in metabolic disturbances, contributing to conditions such as MASLD in hypothyroid states or elevated liver enzymes in hyperthyroidism [11]. Furthermore, liver dysfunction, such as cirrhosis or hepatitis, can impair thyroid hormone metabolism, thereby emphasizing the need for a comprehensive understanding of this dynamic interplay in clinical practice [12].

Thus, a two-sided view of the thyroid–liver interaction is needed. In the following, these two perspectives are analyzed based on the current literature—from the perspective of the thyroid as well as from the perspective of the liver.

## Hypothyroidism and the liver

Hypothyroidism can lead to metabolic derangements that predispose individuals to MASLD [1, 11]. While the connection between thyroid dysfunction and MASLD remains a subject of ongoing research, emerging evidence increasingly supports the hypothesis that thyroid dysfunction contributes to MASLD development.

Metabolic dysfunction-associated steatotic liver disease is defined by hepatic fat accumulation in the absence of alcohol consumption or other known liver disease [10]. Based on this definition, a wide spectrum of pathological disorders fall under the MASLD umbrella characterized by macro-vesicular fat accumulation with/or without hepatocellular injury ± inflammation (MASH; formerly known as NASH) and a degree of fibrosis up to cirrhosis and hepatocellular carcinoma (HCC) [10]. One of the most prevalent chronic liver diseases worldwide, MASLD is estimated to affect approximately 32.4% of the global population [13]. According to the Global Burden of Disease (GBD), it is one of the fastest growing contributors to liver cirrhosis and cancer [9, 14].

The prevalence of MASLD is notably higher in patients with metabolic syndrome, a condition that includes insulin resistance, type 2 diabetes mellitus, dyslipidemia, and central obesity [15]. In contrast, MASH, an advanced form of MASLD, may progress to advanced liver disease, including cirrhosis, liver failure, and hepatocellular carcinoma [16]. Although cirrhosis was traditionally considered irreversible, accumulating evidence suggests that fibrosis, cirrhosis, and even portal hypertension may partially regress following successful treatment of the underlying disease.

**Fig. 2:**
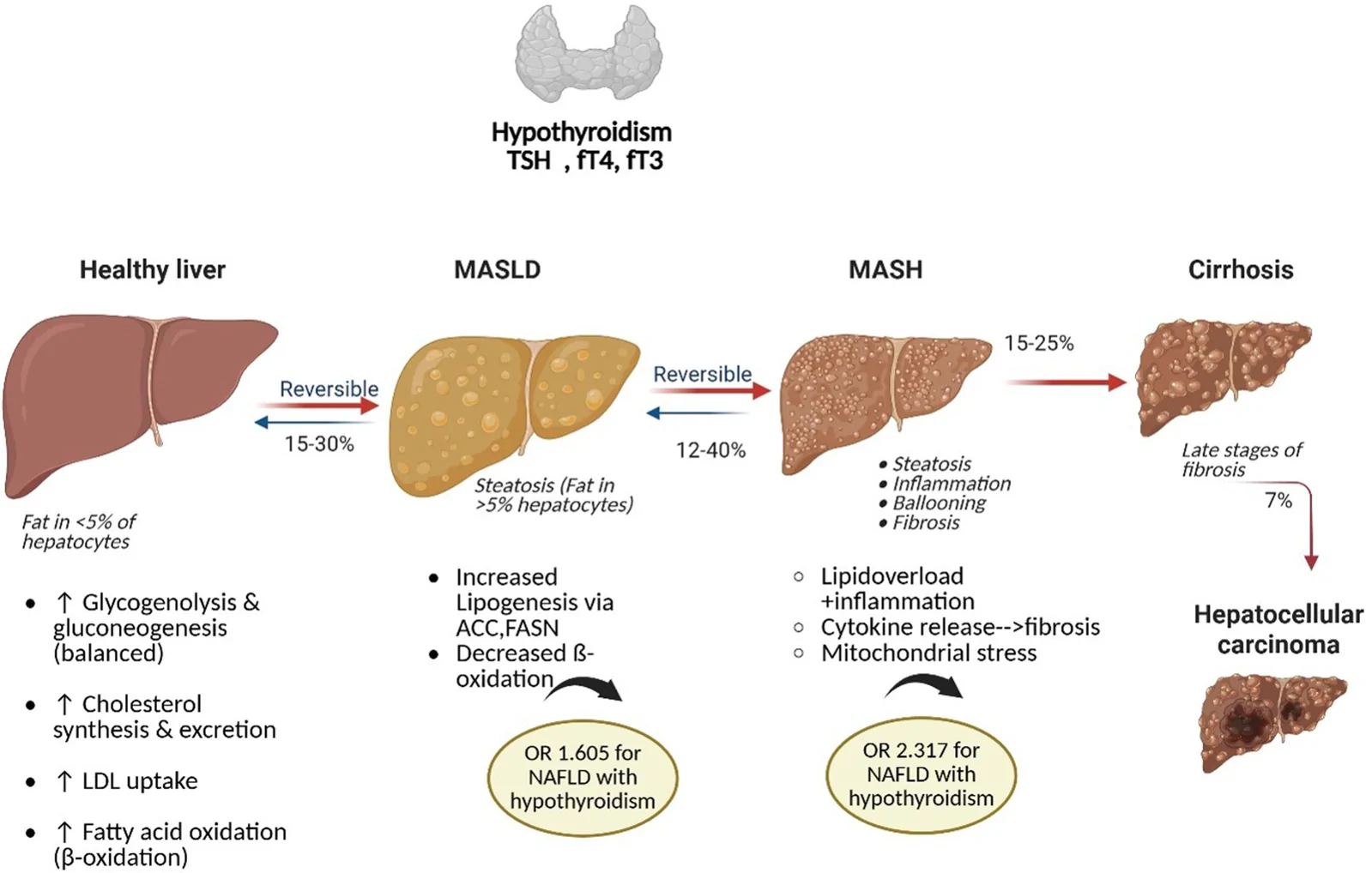
Impact of hypothyroidism on liver disease progression. Progression from healthy liver to metabolic dysfunction-associated steatotic liver disease (*MASLD*), metabolic dysfunction-associated steatohepatitis (*MASH*), cirrhosis, and hepatocellular carcinoma, with the influence of hypothyroidism. Thyroid dysfunction contributes to hepatic steatosis through increased lipogenesis and reduced β‑oxidation, thereby increasing the risk of MASLD (formerly known as NAFLD) and MASH (formerly known as NASH). Created by the authors based on data from [17] *Abbreviations:* TSH - thyroid-stimulating hormone, fT4 - free thyroxine, fT3 free triiodothyronine, LDL  - low density lipoprotein, ACC - acetyl-CoA carboxylase, FASN - fatty acid synthase

As discussed in the section on thyroid–liver physiology, thyroid hormones play a vital role in regulating lipid metabolism in the liver. Any hormonal imbalance can lead to up- or downregulation of metabolism [16]. A systemic review and metanalysis involving 61,548 participants found that patients with MASLD had significantly higher TSH levels as compared to non-MASLD patients. Even higher TSH levels were observed in patients with MASH [10]. Moreover, unclassified hypothyroidism was associated with a higher risk of MASLD (odds ratio [OR] = 1605) and an even greater risk of MASH (OR = 2317; [17]; Fig. 2).

Interestingly, thyroid hormone levels show a dose-dependent effect on the risk of MASLD. A 1 ng/dL increase in fT4 level was associated with a 10.56% reduction in the MASLD risk. In contrast, individuals with higher fT3 levels had a greater prevalence of MASLD compared to those with lower fT3 levels (OR 1.58) [16]. However, these findings represent observational associations and do not necessarily imply a causal relationship. The seemingly divergent associations between fT4 and fT3 may reflect differences in peripheral thyroid hormone metabolism and conversion rather than directly opposing biological effects. Further studies should investigate whether the T3/T4 ratio or altered deiodinase activity contributes to the MASLD risk and progression.

Despite emerging research, a lack of sufficient epidemiological studies on the accurate prevalence of MASLD in euthyroid or hypothyroid patients remains in German-speaking regions like Austria or Germany. A study by Labenz et al., however, highlighted the relationship between hypothyroidism, autoimmune thyroiditis, and the likelihood of MASLD development [18]. This large case–control study matched 57,483 MASLD patients with an equal number of patients without liver disease sourced from the German Disease Analyzer Database. It found that hypothyroidism was associated with a 17% higher risk of MASLD (OR 1.17). Patients with autoimmune thyroiditis showed a 53% higher risk (OR 1.53) of MASLD. Conversely, hyperthyroidism was linked to a lower risk of MASLD (OR 0.85). The increased risk of MASLD in patients with hypothyroidism was consistent in both men and women [18].

## Therapeutic potential of thyroid analogues

There are multiple therapeutic approaches for MASLD, ranging from lifestyle and dietary interventions to bariatric procedures and pharmacological therapies [19]. A recent network meta-analysis comparing incretin-based therapies, fibroblast growth factor 21 (FGF-21) analogues, pan-peroxisome proliferator-activated receptor (pan-PPAR) agonists, and thyroid hormone receptor‑β (THR-β) agonists included 27 studies involving 5357 patients with MASLD/MASH [20]. The analysis demonstrated that incretin-based therapies were most effective in reducing hepatic steatosis, whereas THR‑β agonists showed the greatest effect on fibrosis improvement ([20]; Table 1).

**Table 1:** Current status of therapeutic options in MASLD/MASH

Therapeutic Class	Representative Agents		Current Status
THR‑β agonist	Resmetirom	↓Hepatic steatosis, fibrosis improvement, MASH resolution	MAESTRO-NASH phase III trial; FDA approved; EMA approved
Incretin based therapies	Semaglutide (GLP-1-RA)	Weight loss, MASH resolution, and fibrosis improvement	Positive phase III ESSENCE trial; FDA approved
	Tirzepatide (dual GIP/GLP-1-RA)	Weight loss, ↓hepatic steatosis, fibrosis improvement	Positive phase II SYNERGY-NASH trial; phase III ongoing
	Survodutide (dual GLP1 and glucagon RA)	MASH resolution and fibrosis improvement	Positive phase II outcomes; LIVERAGE phase III ongoing
FGF-21 analogues	Efruxifermin	Liver-directed metabolic, anti-inflammatory, and antifibrotic effects	Positive phase II data; SYMMETRY trial in compensated MASH cirrhosis and SYNCHRONY phase III program ongoing
Pan-PPAR agonist	Lanifibranor	Metabolic, anti-inflammatory, and antifibrotic effects	NATiV3 phase III program ongoing

*Abbreviations: THR‑β* thyroid hormone receptor beta, *GLP-1-RA* glucagon-like peptide‑1 receptor agonist, *FGF*-21 fibroblast growth factor 21, *PPAR* peroxisome proliferator-activated receptor

Recent clinical trials have expanded the therapeutic landscape of MASLD/MASH. Incretin-based therapies, including semaglutide, tirzepatide, and survodutide, have demonstrated significant reductions in hepatic steatosis and bodyweight, together with histological improvements in MASH. Notably, semaglutide has shown positive phase III results and has received regulatory approval, while tirzepatide and survodutide have demonstrated promising phase II outcomes, with evidence of fibrosis improvement [21–24].

For MASH, FGF-21 analogue therapy remains a promising therapeutic approach. Pegozafermin has demonstrated improvements in fibrosis and metabolic parameters in clinical trials. More recently, the phase IIb SYMMETRY trial evaluated efruxifermin in patients with compensated MASH cirrhosis. Although the primary endpoint of fibrosis improvement without worsening of MASH was not achieved at week 36, longer-term follow-up demonstrated numerically higher rates of fibrosis improvement at week 96, particularly in patients receiving the 50-mg dose. These findings suggest a potential benefit of prolonged treatment in advanced MASH, although further confirmation in phase III studies is required [25].

Given the growing evidence linking thyroid dysfunction to MASLD, thyroid analogues have gained increasing interest as potential therapeutic options for reversing liver damage and treating the disease [20, 26]. Preclinical research has shown that TG68 (prodrug) and IS25 (active compound) promote hepatocyte proliferation without overt systemic toxicity in animal models [27].

Following partial liver resection, thyroid hormones have been shown to enhance liver regeneration through activation of the β‑catenin pathway via protein kinase A (PKA) and Wnt signaling [28]. One such promising therapeutic strategy involves the use of THR‑β agonists such as resmetirom, an orally active drug that has demonstrated efficacy in reducing fibrosis and enhancing hepatic fat metabolism [29].

Resmetirom was approved by the FDA under the brand name Rezdiffra™ (Madrigal Pharmaceuticals, Inc., West Conshohocken, Pa, USA) and subsequently received marketing authorization from the EMA in August 2025, based on data from the phase III MAESTRO-NASH trial. In this study, resmetirom demonstrated significant efficacy in achieving MASH resolution and improving liver fibrosis, accompanied by improvements in the NAFLD Activity Score (NAS) after 52 weeks of treatment ([30]; Table 2).

**Table 2:** Key clinical aspects of resmetirom (THR‑β agonist)

*Drug class*
Selective thyroid hormone receptor‑β (THR-β) agonist
*Approval*
FDA 2024 and EMA 2025 for MASH with fibrosis (F2-F3)
*Indication*
Adults with metabolic dysfunction-associated steatohepatitis (MASH) and moderate-to-advanced fibrosis
*Adverse effects*
Common $(\geq 5\mathrm{{\%}})$: diarrhea, nausea, pruritus, vomiting, constipation, abdominal pain, dizziness
Warnings: hepatotoxicity (monitor LFTs, bilirubin), gallbladder-related events (cholelithiasis, ...)
Overall: generally well tolerated; most events mild to moderate
*Laboratory monitoring*
Liver biochemistry + bilirubin (for hepatotoxicity)
Thyroid function (TSH/fT4/fT3)
Lipid panel (LDL, TG, ApoB)
Noninvasive liver imaging/stiffness (MRI, elastography)
*Drug interaction*
↑ Statin exposure → limit dose, monitor for myopathy and LFT elevations
Strong CYP2C8 inhibitors (e.g., gemfibrozil) → avoid
OATP1B1/1B3 inhibitors (e.g., cyclosporine) → contraindicated
*Limited uses/precautions*
Not indicated in MASLD without steatohepatitis
Not recommended in cirrhosis (F4) or moderate-severe hepatic impairment (Child–Pugh B/C)

Created with BioRender.com and published under a BioRender Publication License

Although the safety profile is generally acceptable, with mild gastrointestinal side effects being the most common toxicity, long-term monitoring is necessary to assess possible risks related to thyroid function, gonadal health, and bone metabolism [30]. A recent real-world study published in April 2025 reported that resmetirom adverse events were observed in about 40%, and approximately 16% discontinued therapy, primarily due to gastrointestinal or dermatologic side effects [31]. There was no drug-induced liver injury detected. These findings suggest that although resmetirom is generally well tolerated in clinical trials, tolerability in clinical practice may be lower, emphasizing the need for close follow-up [31]. Clinical implementation of resmetirom also faces challenges related to patient selection and treatment response monitoring, which rely on noninvasive liver fibrosis tests. Nonetheless, resmetirom represents a significant advance in the treatment of MASLD and MASH, paving the way for future therapies targeting this complex and often-progressive disease.

Beyond their metabolic and antifibrotic effects, thyroid hormones and their receptors have also been implicated in hepatocarcinogenesis. The complex role of thyroid hormone signaling in HCC is discussed in a separate section below.

## Hyperthyroidism and the liver

Hyperthyroidism has an estimated incidence of 0.05% to 1.3%, predominantly affecting women (female to male ratio approximately 5:1) [32]. In untreated cases, liver dysfunction is common, with abnormal liver biochemistry reported in about 15%–76% of patients [32]. A 2021 meta-analysis by Scappaticcio et al. found that 55%–60% of hyperthyroid patients exhibit at least one abnormal liver function parameter [33, 34].

The underlying mechanisms are multifactorial. Prolonged thyrotoxicosis can result in direct hepatotoxicity due to increased metabolic demand and oxygen consumption, leading to oxidative stress and hepatocyte hypoxia. Additionally, hyperthyroidism promotes accelerated protein and glycogen catabolism in hepatocytes [32].

An autoimmune overlap is well documented, particularly in patients with Graves’ disease, where coexistent autoimmune hepatitis or primary biliary cholangitis (PBC) may exacerbate hepatic dysfunction [35, 36]. Patients with autoimmune liver conditions frequently have concurrent thyroid dysfunction and test positive for antithyroglobulin and antimicrosomal antibodies, suggesting a shared immunogenic predisposition [36]. In addition, treatment-related hepatotoxicity must be considered. For instance, antithyroid drugs, such as methimazole and propylthiouracil, have been associated with hepatotoxic effects ranging from transient asymptomatic transaminase elevations to fulminant hepatitis and cholestasis [37]. Conversely, therapies used in liver disease—including interferon therapy, corticosteroids, and propranolol—can themselves disturb thyroid metabolism, causing thyroid dysfunction [1, 12].

## Impact of liver dysfunction on thyroid function

Given the strong clinical association between the liver and thyroid dysfunction, it is critical to monitor serum thyroid hormone levels carefully in patients with liver disease. Routine thyroid testing can be misleading in patients with liver disease. For instance, in liver dysfunction, particularly in conditions such as cirrhosis, the synthesis of thyroglobulin-binding globulin (TBG) can be altered, leading to changes in free thyroid hormone levels and potentially causing inaccurate thyroid function tests [1].

Chronic liver diseases, particularly cirrhosis, can result in “euthyroid sick syndrome,” also known as non-thyroidal illness syndrome (NTIS), where thyroid hormone levels may be altered in the absence of intrinsic thyroid disease. In this syndrome, patients may exhibit low T3 levels with normal or low levels of T4 and normal or low TSH, with the low T3 levels reflecting impaired peripheral conversion due to liver dysfunction [38].

## Possible prognostic markers in liver disease

Considering all these alterations, thyroid function tests may serve as prognostic markers in acute and chronic liver disease. Recent evidence suggests that declining fT3 levels are associated with increasing disease severity in patients with advanced chronic liver disease (ACLD) as well as with a higher risk of hepatic decompensation, acute-on-chronic liver failure (ACLF), and liver-related mortality [39]. These findings support the potential value of thyroid hormone parameters, particularly fT3, as indicators of disease progression and prognosis. A prospective cohort study explored the role of free triiodothyronine in predicting the 90-day prognosis of patients with hepatitis B virus (HBV)-related acute-on-chronic liver failure [40]. Findings showed that 80% of the 122 patients with HBV-related acute-on-chronic liver failure had decreased fT3 levels, with the lowest fT3 results occurring roughly 8.5 days after admission and lasting about 16 days. The fT3 level, fT3 change type, and hepatic encephalopathy (HE) grading were the most significant factors influencing prognosis. The formula developed based on these factors demonstrated high predictive accuracy (AUC = 0.892), surpassing conventional scoring systems like CTP, MELD, MELD-Na, CLIF-SOFA, and AARC scores [40]. This highlights fT3’s potential as a valuable prognostic marker for short-term survival. Furthermore, a study by Feng et al. [41] supported the finding that NTIS commonly develops in patients with liver failure, highlighting its clinical significance in the context of thyroid–liver interactions [41]. These findings further support the potential value of fT3-based prognostic models for assessing short-term mortality in patients with liver failure.

Additionally, a study investigating TSH as a prognostic marker in chronic liver disease reported higher TSH levels in non-survivors compared to survivors, whereas T3 and fT4 levels showed no independent prognostic value [42]. The role of TSH in cirrhosis appears to be compensatory, possibly maintaining thyroid homeostasis as liver function deteriorates [43].

## Role of thyroid hormones in hepatocellular carcinoma

Hepatocellular carcinoma represents a significant global health challenge, particularly in advanced stages of the disease [44]. Emerging evidence suggests that thyroid hormone signaling may play an important role in hepatocarcinogenesis, tumor progression, and metabolic reprogramming of cancer cells. Alterations in thyroid hormone receptors (TRs), including reduced receptor expression and receptor dysfunction, have been implicated in the development and progression of HCC [3].

Preclinical studies have shown that T3 reactivates the thyroid hormone receptor beta (THR-β) axis, promoting a shift from an aggressive tumor cell toward a more differentiated type. Thyroid hormone receptor beta exhibits antiproliferative effects in cancer cells and acts as a differentiation factor [45]. This effect involves genomic reprogramming, a metabolic transition from aerobic glycolysis to oxidative metabolism, and tumor growth suppression [46]. Aditionally, T3 and thyromimetic compounds like TG68 result in reduced HCC progression. Studies have shown this to be via reversing paraneoplastic changes, enhancing fatty acid oxidation, and downregulating oncogenes [47].

Recent experimental evidence has further strengthened the potential role of thyroid hormone signaling in HCC. A 2025 study demonstrated that T3 inhibits HCC progression and enhances the antitumor activity of lenvatinib by modulating glucose metabolism through thyroid hormone-responsive spot 14 homolog (THRSP)-mediated signaling pathways, thereby suppressing glycolysis and tumor growth [48]. These findings suggest that thyroid hormone signaling may influence not only tumor biology but also therapeutic responsiveness.

In addition to their direct effects on tumor cells, thyroid hormones are closely linked to MASLD, a major risk factor for HCC development. Therefore, modulation of thyroid hormone signaling may have implications both for prevention of HCC through improvement of underlying liver disease and in terms of potential therapeutic intervention in established cancer.

Despite these promising findings, the role of thyroid hormone signaling in HCC remains incompletely understood. While preclinical studies support antiproliferative and tumor-suppressive effects of THR-β activation, whether these observations can be translated into meaningful clinical benefit remains uncertain. Likewise, the long-term impact of THR‑β agonists such as resmetirom on HCC development has not yet been established and warrants further investigation. At present, thyroid hormone signaling should be regarded as a promising area of research rather than as an established therapeutic strategy for HCC.

## Closing statement

This article highlights the intertwined relationship between thyroid and liver metabolism, emphasizing their profound interdependence. The emerging clinical evidence, along with the emergence of newly approved therapeutic agents, underscores the significance of this axis. Optimal management of patients with MASLD requires an interdisciplinary approach involving physicians, dietitians, and pharmacists, aiming not only at disease prevention but also at offering effective treatments for enhanced long-term outcomes. Special attention must be given to the physiological interplay between thyroid dysfunction and liver disease, as it may critically influence disease progression and the treatment response.

There remains considerable therapeutic promise, particularly in the fields of HCC and liver cirrhosis. Substantially, MASLD continues to be unacknowledged among patients, who often underestimate its seriousness. It is crucial to raise awareness that MASLD, like other chronic liver diseases, demands close follow-up and timely intervention, especially in patients with coexisting thyroid dysfunction.

## Data Availability

All data supporting the findings of this work are included in the article.

## References

[1] Huang MJ, Liaw YF. Clinical associations between thyroid and liver diseases. J Gastroenterol Hepatol. 1995;10(3):344–50. 10.1111/j.1440-1746.1995.tb01106.x. 7548816

[2] Malik R. The relationship between the thyroid gland and the liver. QJM. 2002;95(9):559–69. 10.1093/qjmed/95.9.559. 12205333

[3] Tang Q, Zeng M, Chen L, Fu N. Targeting Thyroid Hormone/Thyroid Hormone Receptor Axis: An Attractive Therapy Strategy in Liver Diseases. Front Pharmacol. 2022;13:871100. 10.3389/fphar.2022.871100. PMC9201453 35721201

[4] Karlsen TH, Hutchinson SJ, Zelber-Sagi S, Carrieri P, Negro F, Lim AG, et al. Implementing sustainable liver health in Europe: a second EASL-Lancet Commission. Lancet. 2026;407(10541):1825–90. 10.1016/S0140-6736(26)00138-8. 42068985

[5] Marino L, Kim A, Ni B, Celi FS. Thyroid hormone action and liver disease, a complex interplay. Hepatology. 2025;81(2):651–69. 10.1097/HEP.0000000000000551. PMC11737129 37535802

[6] Sinha RA, Bruinstroop E, Yen PM. Actions of thyroid hormones and thyromimetics on the liver. Nat Rev Gastroenterol Hepatol. 2025; (1):9-22. 10.1038/s41575-024-00991-4. Epub 2024 Oct 17. PMC7616774 39420154

[7] Sinha RA. Thyroid Hormone-Mediated Selective Autophagy and Its Implications in Countering Metabolic Dysfunction-Associated Steatotic Liver Disease. Endocrinol Metab. 2024;39(5):686–92. 10.3803/EnM.2024.2068. PMC11525703 39397515

[8] Soares De Oliveira L, Ritter MJ. Thyroid hormone and the Liver. Hepatol Commun. 2024; 9(1):e0596. 10.1097/hc9.0000000000000596. eCollection 2025 Jan 1. PMC11661762 39699315

[9] Riazi K, Azhari H, Charette JH, Underwood FE, King JA, Afshar EE, et al. The prevalence and incidence of NAFLD worldwide: a systematic review and meta-analysis. Lancet Gastroenterol Hepatol. 2022;7(9):851–61. 10.1016/S2468-1253(22)00165-0. 35798021

[10] Kleiner DE, Brunt EM, Van Natta M, Behling C, Contos MJ, Cummings OW, et al. Design and validation of a histological scoring system for nonalcoholic fatty liver disease. Hepatology. 2005;41(6):1313–21. 10.1002/hep.20701. 15915461

[11] Tanase DM, Gosav EM, Neculae E, Costea CF, Ciocoiu M, Hurjui LL, et al. Hypothyroidism-Induced Nonalcoholic Fatty Liver Disease (HIN): Mechanisms and Emerging Therapeutic Options. International Journal of Molecular Sciences. 2020;21(16)5927. 10.3390/ijms21165927. PMC7460638 32824723

[12] Punekar P, Sharma AK, Jain A. A Study of Thyroid Dysfunction in Cirrhosis of Liver and Correlation with Severity of Liver Disease. Indian J Endocrinol Metab. 2018;22(5):645–50. 10.4103/ijem.IJEM_25_18. PMC6166553 30294575

[13] Quek J, Chan KE, Wong ZY, Tan C, Tan B, Lim WH, et al. Global prevalence of non-alcoholic fatty liver disease and non-alcoholic steatohepatitis in the overweight and obese population: a systematic review and meta-analysis. Lancet Gastroenterol Hepatol. 2023;8(1):20–30. 10.1016/S2468-1253(22)00317-X. 36400097

[14] Younossi ZM, Golabi P, Paik JM, Henry A, Van Dongen C, Henry L. The global epidemiology of nonalcoholic fatty liver disease (NAFLD) and nonalcoholic steatohepatitis (NASH): a systematic review. Hepatology. 2023;77(4):1335–47. 10.1097/HEP.0000000000000004. PMC10026948 36626630

[15] En Li Cho E, Ang CZ, Quek J, Fu CE, Lim LKE, Heng ZEQ, et al. Global prevalence of non-alcoholic fatty liver disease in type 2 diabetes mellitus: an updated systematic review and meta-analysis. Gut. 2023;72(11):2138–48. 10.1136/gutjnl-2023-330110. 37491159

[16] Xiang LL, Cao YT, Sun J, Li RH, Qi F, Zhang YJ, et al. Association between thyroid function and nonalcoholic fatty liver disease: a dose-response meta-analysis. Front Endocrinol. 2024;15:1399517. 10.3389/fendo.2024.1399517. PMC11231071 38982990

[17] Guo Z, Li M, Han B, Qi X. Association of non-alcoholic fatty liver disease with thyroid function: A systematic review and meta-analysis. Dig Liver Dis. 2018;50(11):1153–62. 10.1016/j.dld.2018.08.012. 30224316

[18] Labenz C, Kostev K, Armandi A, Galle PR, Schattenberg JM. Impact of thyroid disorders on the incidence of non-alcoholic fatty liver disease in Germany. United European Gastroenterol J. 2021;9(7):829–36. 10.1002/ueg2.12124. PMC8435260 34288580

[19] Roeb E. MASLD – Krankheitslast, Herausforderungen und neue Therapeutika. Gastroenterologie. 2025;20(2):80–93. 10.1007/s11377-025-00868-1.

[20] Lin RT, Sun QM, Xin X, Ng CH, Valenti L, Hu YY, et al. Comparative efficacy of THR‑β agonists, FGF-21 analogues, GLP-1R agonists, GLP-1-based polyagonists, and Pan-PPAR agonists for MASLD: A systematic review and network meta-analysis. Metabol Open. 2024;161:156043. 10.1016/j.metabol.2024.156043. 39357599

[21] Hughes JW, Levin JB, Sajatovic M, Sclair SN. Semaglutide for metabolic dysfunction-associated steatohepatitis (MASH): Estimating eligibility from the 2021-2023 National Health and Nutrition Examination Survey (NHANES). Hepatol Commun. 2025;9(12)e0860. 10.1097/HC9.0000000000000860. PMC12668583 41325108

[22] Sanyal AJ, Newsome PN, Kliers I, Østergaard LH, Long MT, Kjær MS, et al. Phase 3 Trial of Semaglutide in Metabolic Dysfunction–Associated Steatohepatitis. N Engl J Med. 2025;392(21):2089–99. 10.1056/NEJMoa2413258. 40305708

[23] Loomba R, Hartman ML, Lawitz EJ, Vuppalanchi R, Boursier J, Bugianesi E, et al. Tirzepatide for Metabolic Dysfunction-Associated Steatohepatitis with Liver Fibrosis. N Engl J Med. 2024;391(4):299–310. 10.1056/NEJMoa2401943. 38856224

[24] Sanyal AJ, Bedossa P, Fraessdorf M, Neff GW, Lawitz E, Bugianesi E, et al. A Phase 2 Randomized Trial of Survodutide in MASH and Fibrosis. N Engl J Med. 2024;391(4):311–9. 10.1056/NEJMoa2401755. 38847460

[25] Noureddin M, Frias JP, Neff GW, Lucas KJ, Behling C, Bedossa P, et al. Safety and efficacy of once-weekly efruxifermin versus placebo in metabolic dysfunction-associated steatohepatitis (HARMONY): 96-week results from a multicentre, randomised, double-blind, placebo-controlled, phase 2b trial. Lancet. 2025;406(10504):719–30. 10.1016/S0140-6736(25)01073-6. 40818852

[26] Wang W, Gao X, Niu W, Yin J, He K. Targeting Metabolism: Innovative Therapies for MASLD Unveiled. International Journal of Molecular Sciences. 2025;26(9)4077. 10.3390/ijms26094077. PMC12071536 40362316

[27] Perra A, Kowalik MA, Cabras L, Runfola M, Sestito S, Migliore C, et al. Potential role of two novel agonists of thyroid hormone receptor‑β on liver regeneration. Cell Prolif. 2020;53(5)e12808. 10.1111/cpr.12808. PMC7260063 32347601

[28] Alvarado TF, Puliga E, Preziosi M, Poddar M, Singh S, Columbano A, et al. Thyroid Hormone Receptor β Agonist Induces β‑Catenin-Dependent Hepatocyte Proliferation in Mice: Implications in Hepatic Regeneration. Gene Expr. 2016;17(1):19–34. 10.3727/105221616X691631. PMC5215473 27226410

[29] Harrison SA, Bashir MR, Guy CD, Zhou R, Moylan CA, Frias JP, et al. Resmetirom (MGL-3196) for the treatment of non-alcoholic steatohepatitis: a multicentre, randomised, double-blind, placebo-controlled, phase 2 trial. Lancet. 2019;394(10213):2012–24. 10.1016/S0140-6736(19)32517-6. 31727409

[30] Petta S, Targher G, Romeo S, Pajvani UB, Zheng MH, Aghemo A, et al. The first MASH drug therapy on the horizon: Current perspectives of resmetirom. Liver Int. 2024;44(7):1526–36. 10.1111/liv.15930. 38578141

[31] Ravela N, Shackelford P, Blessing N, Yoder L, Chalasani N, Samala N. Early experience with resmetirom to treat metabolic dysfunction-associated steatohepatitis with fibrosis in a real-world setting. Hepatol Commun. 2025;9(4)e0670. 10.1097/HC9.0000000000000670. PMC11970881 40178494

[32] Yorke E. Hyperthyroidism and Liver Dysfunction: A Review of a Common Comorbidity. Clin Med Insights Endocrinol Diabetes. 2022;15:11795514221074672. 10.1177/11795514221074672. PMC8829710 35153522

[33] Scappaticcio L, Longo M, Maiorino MI, Pernice V, Caruso P, Esposito K, et al. Abnormal Liver Blood Tests in Patients with Hyperthyroidism: Systematic Review and Meta-Analysis. Thyroid. 2021;31(6):884–94. 10.1089/thy.2020.0715. 33327837

[34] Kubota S, Amino N, Matsumoto Y, Ikeda N, Morita S, Kudo T, et al. Serial changes in liver function tests in patients with thyrotoxicosis induced by Graves’ disease and painless thyroiditis. Thyroid. 2008;18(3):283–7. 10.1089/thy.2007.0189. 18001177

[35] Shetty S, Rajasekaran S, Venkatakrishnan L. Grave’s Disease and Primary Biliary Cirrhosis-An Unusual and Challenging Association. J Clin Exp Hepatol. 2014;4(1):66–7. 10.1016/j.jceh.2013.08.001. PMC4017187 25755537

[36] Elta GH, Sepersky RA, Goldberg MJ, Connors CM, Miller KB, Kaplan MM. Increased incidence of hypothyroidism in primary biliary cirrhosis. Dig Dis Sci. 1983;28(11):971–5. 10.1007/BF01311724. 6628157

[37] Khan AA, Ata F, Aziz A, Elamin H, Shahzad A, Yousaf Z, et al. Clinical Characteristics and Outcomes of Patients With Antithyroid Drug-Related Liver Injury. J Endocr Soc. 2023;8(1)bvad133. 10.1210/jendso/bvad133. PMC10765380 38178906

[38] Nardin GD, da S Colombo B, Ronsoni MF, Silva PESE, Fayad L, Wildner LM, et al. Thyroid hormone profile is related to prognosis in acute decompensation of cirrhosis. Arch Endocrinol Metab. 2024;68:e230249. 10.20945/2359-4292-2023-0249. PMC11460973 39420934

[39] Hartl L, Simbrunner B, Jachs M, Wolf P, Bauer DJM, Scheiner B, et al. Lower free triiodothyronine (fT3) levels in cirrhosis are linked to systemic inflammation, higher risk of acute-on-chronic liver failure, and mortality. JHEP Rep. 2024;6(1)100954. 10.1016/j.jhepr.2023.100954. PMC10733101 38125301

[40] Zhang J, Chen Y, Ding M, Duan Z. Correlation between dynamic changes in free triiodothyronine levels and 90-day prognosis in patients with HBV-related acute-on-chronic liver failure. Eur J Med Res. 2022;27(1)88. 10.1186/s40001-022-00718-8. PMC9175361 35672864

[41] Feng HL, Li Q, Cao WK, Yang JM. Changes in thyroid function in patients with liver failure and their clinical significance: A clinical study of non-thyroidal illness syndrome in patients with liver failure. Hepatobiliary Pancreat Dis Int. 2020;19(6):561–6. 10.1016/j.hbpd.2020.05.001. 32535064

[42] Belu AM, Nicoara AD, Belu DM, Circo E. Evaluation of MELD Scores and Thyroid Hormones as Prognostic Factors of Liver Cirrhosis. Medicina. 2024;60(9)1474. 10.3390/medicina60091474. PMC11433705 39336515

[43] Wu Y, You S, Zang H, Liu H, Mao Y, Mao P, et al. Usefulness of serum thyroid-stimulation hormone (TSH) as a prognostic indicator for acute-on-chronic liver failure. Ann Hepatol. 2015;14(2):218–24. 25671831

[44] Mauro E, De Castro T, Zeitlhoefler M, Sung MW, Villanueva A, Mazzaferro V, et al. Hepatocellular Carcinoma: Epidemiology, diagnosis and treatment. JHEP Reports. 2025 Sep;101571. https://doi.org/10.1016/j.jhepr.2025.101571 10.1016/j.jhepr.2025.101571 PMC12615749 41244300

[45] Gionfra F, De Vito P, Pallottini V, Lin HY, Davis PJ, Pedersen JZ, et al. The Role of Thyroid Hormones in Hepatocyte Proliferation and Liver Cancer. Front Endocrinol. 2019;10:532. 10.3389/fendo.2019.00532. PMC6730500 31543862

[46] Kowalik MA, Puliga E, Cabras L, Sulas P, Petrelli A, Perra A, et al. Thyroid hormone inhibits hepatocellular carcinoma progression via induction of differentiation and metabolic reprogramming. J Hepatol Suppl. 2020;72(6):1159–69. 10.1016/j.jhep.2019.12.018. 31954205

[47] Caddeo A, Serra M, Sedda F, Bacci A, Manera C, Rapposelli S, et al. Potential use of TG68 - A novel thyromimetic - for the treatment of non-alcoholic fatty liver (NAFLD)-associated hepatocarcinogenesis. Front Oncol. 2023;13:1127517. 10.3389/fonc.2023.1127517. PMC9996294 36910628

[48] Yang CC, Yan YC, Pan GQ, Meng GX, Zhang X, Yan LJ, et al. Thyroid hormones inhibit tumor progression and enhance the antitumor activity of lenvatinib in hepatocellular carcinoma via reprogramming glucose metabolism. Cell Death Discov. 2025;11(1)92. 10.1038/s41420-025-02378-z. PMC11889155 40055330

